# Prospective phase II study of preoperative short-course radiotherapy for rectal cancer with twice daily fractions of 2.9 Gy to a total dose of 29 Gy - Long-term results

**DOI:** 10.1186/1748-717X-4-67

**Published:** 2009-12-21

**Authors:** Matthias Guckenberger, Joern Wulf, Andreas Thalheimer, Daniel Wehner, Arnulf Thiede, Gottfried Müller, Marco Sailer, Michael Flentje

**Affiliations:** 1 Department of Radiation Oncology, University of Würzburg, Würzburg, Germany; 2 Department of Radiooncology, Lindenhofspital, Bern, Switzerland; 3 Department of Surgery, University of Würzburg, Würzburg, Germany; 4 Department of Surgery, Caritas-Krankenhaus, Bad Mergentheim, Germany; 5 Department of Surgery, Bethesda - AK Bergedorf, Hamburg, Germany

## Abstract

**Background:**

To evaluate clinical outcome after preoperative short-course radiotherapy for rectal cancer with twice daily fractions of 2.9 Gy to a total dose of 29 Gy and adjuvant chemotherapy for pathological stage UICC ≥ II.

**Methods:**

118 patients (median age 64 years; male : female ratio 2.5 : 1) with pathological proven rectal cancer (clinical stage II 50%, III 41.5%, IV 8.5%) were treated preoperatively with twice daily radiotherapy of 2.9 Gy single fraction dose to a total dose of 29 Gy; surgery was performed immediately in the following week with total mesorectal excision (TME). Adjuvant 5-FU based chemotherapy was planned for pathological stage UICC ≥ II.

**Results:**

After low anterior resection (70%) and abdominoperineal resection (30%), pathology showed stage UICC I (27.1%), II (25.4%), III (37.3%) and IV (9.3%). Perioperative mortality was 3.4% and perioperative complications were observed in 22.8% of the patients. Adjuvant chemotherapy was given in 75.3% of patients with pathological stage UICC ≥ II. After median follow-up of 46 months, five-year overall survival was 67%, cancer-specific survival 76%, local control 92% and freedom from systemic progression 75%. Late toxicity > grade II was observed in 11% of the patients.

**Conclusions:**

Preoperative short-course radiotherapy, total mesorectal excision and adjuvant chemotherapy for pathological stage UICC ≥ II achieved excellent local control and favorable survival.

## Background

Multimodality treatment for rectal cancer is well established for more than 20 years after adjuvant radiochemotherapy has shown to improve overall survival [1]. Significant progress has been made in surgical, radiation and medical therapy: total mesorectal excision (TME) has become a surgical standard [2] and neoadjuvant radiotherapy (± chemotherapy) improved outcome compared to postoperative treatment.

However, there is considerable debate regarding the best approach to preoperative therapy. The CAO/ARO/AIO-94 trial reported improved local control with decreased toxicity after preoperative compared to postoperative radiochemotherapy [3]: this so-called long-course treatment delivers conventionally fractionated radiotherapy of 45 Gy to 50 Gy and delayed surgery is performed to allow for tumor regression. So-called short-course preoperative radiotherapy with 5 fractions of 5 Gy and immediate surgery has shown to improve survival compared to surgery alone in the pre-TME era [4]. Combined with documented quality controlled TME-surgery, short-course preoperative radiotherapy decreased rates of local recurrence, however, no survival benefit was observed [5]. A recent update of this Dutch TME trial confirmed the benefit regarding local control; however, systemic disease progression limited survival. One Polish study compared preoperative short-course radiotherapy with preoperative long-course radiochemotherapy and no differences in survival, local control, late toxicity and quality of life were observed [6,7].

We report on a phase II study for rectal cancer clinical stage UICC ≥ II with neoadjuvant short-course radiotherapy followed by immediate TME surgery. The protocol differs in two important aspects form the original Swedish and Dutch protocol. Because the high single fraction dose of 5 Gy remains an issue of concern in short-course preoperative radiotherapy, a modification of the 5 × 5 Gy fractionation was introduced: a total dose of 29 Gy was delivered with twice daily fractions of 2.9 Gy; an interval of at least 6 hours between the daily fractions was mandatory to allow recovery of normal tissue and the total treatment time of one week was maintained. Additionally, adjuvant chemotherapy was indicated in cases of pathological stage UICC ≥ II aiming at decreasing rates of systemic progression. This article reports the long-term clinical results of the 118 patients treated between 2000 and 2007.

## Methods

### Eligibility Criteria

It was the aim of this trial, to evaluate the clinical outcome of short-course preoperative radiotherapy with adjuvant chemotherapy for rectal cancer with UICC ≥ II in a prospective fashion. Patients with surgery planned at the University Hospital Würzburg or one affiliated academic teaching hospital were eligible for this prospective phase II study. Patients needed to have pathological proven rectal cancer UICC stage II to IV at any age and Karnofsky index > 70. The upper limit of the lower tumor border was 15 cm from the anal verge. Inclusion of stage IV was limited to patients who presented with potentially resectable hepatic metastases.

Neoadjuvant radiochemotherapy was chosen instead of participation in this trial if the surgeon and radiation oncologist expected that downstaging could 1) achieve sphincter preservation in patients with very low tumor location or 2) improve complete resectability in patients with cT4 tumors. Patients with invasion of the sphincter or very low tumor location, where no sufficient downstaging for sphincter preservation after neoadjuvant radiochemotherapy was assumed, were eligible for this trial.

Prior irradiation of the pelvic region and severe comorbidities, which contraindicate adjuvant chemotherapy, were exclusion criteria. All Patients provided written informed consent upon participation and the protocol was approved by the institutional review board of the University Hospital Würzburg.

### Study Design and Treatment

Staging of the patients required colonoscopy of the rectum and entire large bowel and computed tomography of the pelvis, abdomen and chest. Endorectal ultrasound was performed for staging of T and N status; pelvic MRI was not routine practice at the time, when this protocol was developed.

A belly board was used for patient set-up in prone position at radiotherapy treatment planning and delivery. Treatment planning was based on computed tomography. The superior border of the planning target volume (PTV) was the top of the fifth lumbar vertebra in patients with cN+ and/or tumor location in the proximal third of the rectum and the top of the first sacral vertebra in patients with cN- and tumor location in the middle and lower third of the rectum. The inferior border of the PTV was 1 cm below the pelvic floor and the perineum was included if abdominoperineal resection (APR) was planned. In axial directions, the PTV encompassed the rectum, presacral space and the pelvic lymphatics. Radiotherapy was based on three dimensional conformal treatment planning; a three field technique with one posterior field and two wedged lateral fields was applied using 18 MV photon energy for the lateral fields and 6 MV photon energy for the posterior field. A total dose of 29 Gy was prescribed with twice daily fraction doses of 2.9 Gy; interval between the daily fractions was at least 6 hours. Radiotherapy treatment was always delivered within one week starting on Monday to Friday. This total dose of 29 Gy equates the standard protocol of 5 × 5 Gy according the LQ-model with an α/β-ratio of 10 Gy for the tumor to compensate for the lower single fraction dose.

Surgery was planned in the week immediately after radiotherapy. Patients underwent radical resection of the rectal cancer with total mesorectal excision (TME). Surgery was performed at the Department of Surgery of the University Hospital Würzburg (n = 95) or an affiliated academic teaching hospital (n = 22).

Adjuvant chemotherapy was planned for patients with pathological stage UICC ≥ II. 5-Fluorouracil (5-FU) (425 mg/m^2^) as bolus infusion and folinic acid (25 mg/m^2^) was given for five days/cycle and six cycles were planned every 28 days.

Patients were routinely followed up at 6 weeks, every three months for the first two years, every six months for another three years and once annually thereafter. Acute toxicity during treatment and within the first 6 months was scored using the CTC 2.0 and later the CTCAE v3.0; chronic toxicity more than 6 months after treatment was scored with CTCAE v3.0.

### Calculation of biological effective doses

Biological effective doses (BED) were calculated for comparison of different fractionations of radiotherapy. Values of α/β = 10 Gy for the rectal tumor and α/β = 3 Gy for late normal tissue toxicity were used. BED doses for late toxicity were calculated with the formula

where n is the number of fractions and d the single fraction dose (Gy).

For tumor effects, the overall treatment time was taken into account using the formula

with a daily repair rate γ/α of 0.6 Gy, which is a measure for how much dose is lost per day due to tumor tissue repair. A proliferation delay T_k _of 7 days was used (Colorectal Cancer Collaborative Group, 2001), which was subtracted from the overall treatment time T. No tumor tissue repair was assumed for a total treatment time of 5 days and both T and T_k _were set to 0 days.

### Statistics

Survival and recurrence data were calculated by the Kaplan-Meier method using Statistica v7 software (Statsoft, Tulsa, OK, USA). Overall survival (OS), cancer-specific survival (CSS), disease-free survival (DFS), local control (LC) and freedom from systemic progression (FSP) were calculated. Results for different subgroups were compared using the log-rank statistic. Results with p < 0.05 were considered as statistically significant.

## Results

### Treatment and toxicity

Between 2000 and 2007, 118 patients with pathological proven rectal cancer were included into this trial. Median age was 64 years and male/female ratio was 2.5/1. Tumor location was the upper, middle and lower third of the rectum in 8.5%, 50% and 41.5% of the patients, respectively. Ten patients with clinical stage UICC IV were included: 7 patients showed potentially resectable hepatic metastases and three patients with pulmonary metastases violated the exclusion criteria. Pretreatment patient and tumor characteristics are shown in Table 1.

**Table 1:** Baseline characteristics of all 118 patients

Age median/range (years)	64/30 - 84
**Female/male no**.	35/83

**Clinical tumor stage no. (%)**	
cT2	11 (9.3)
cT3	106 (89.3)
cT4	1 (0.8)

**Clinical nodal stage no. (%)**	
Node negative	60 (50,8)
Node positive	54 (45.8)
Unknown	4 (3.4)

**Clinical UICC stage no. (%)**	
II	59 (50)
III	49 (41.5)
IV	10 (8.5)

**Tumor location no. (%)**	
Upper third of rectum	10 (8.5)
Middle third of rectum	59 (50)
Lower third of rectum	49 (41.5)

Radiotherapy was delivered according to protocol in 94.1% of the patients; in 7 patients, radiotherapy was delivered on three days with twice daily 2.9 Gy and two days with a single fraction of 5 Gy. Acute toxicity during radiotherapy was maximum grade I in all patients. The interval between the end of radiotherapy and surgery was three or four days in 89% of the patients. LAR and APR was performed in 68.8% and 29.7% of the patients, respectively. One 75 year old female denied radical surgery and was treated with local excision; one 80 year old female did not receive any surgery because of exacerbation of comorbidities. Pathological examination showed UICC stage I in 27.1% of the patients. Complete local resection of the rectal cancer was achieved in 91.6%, incomplete local resection of the rectal tumor with residual microscopic disease was observed in 5.1% and local resection status was unknown in 2.5%. Systemic disease remained in 3.4% of the patients. Treatment characteristics are listed in Table 2.

**Table 2:** Treatment characteristics

RT according to protocol no. (%)	111 (94.1)
**Interval between end of RT and surgery median/range (days)**	3/3 - 10

**Type of surgery no. (%)**	
LAR	81 (68.6)
APR	35 (29.7)
Local excision	1 (0.8)
No surgery	1 (0.8)
Protective stoma after LAR	72 (87.8)

**Pathological tumor stage no. (%)**	
pT1	8 (6.8)
pT2	34 (28.8)
pT3	74 (62.7)
pT4	1 (0.8)

**Pathological nodal stage no. (%)**	
Node negative	64 (54.2)
Node positive	53 (44.9)

**Pathological UICC stage no. (%)**	
I	32 (27.1)
II	30 (25.4)
III	44 (37.3)
IV	11 (9.3)

**Resection status no. (%)**	
R0	104 (88.1)
R1 (all local)	6 (5.1)
R2 (all systemic)	4 (3.4)
Unknown	3 (2.5)
**Number of removed lymph nodes Median/range**	12/1-30

**Indication for chemotherapy no. (%)**	81 (68.6)

**Actually performed chemotherapy no. (%)**	61 (75.3)
5-FU & LV chemotherapy	35 (57.4)
FOLFOX4	15 (24.6)
others	11 (18)

RT (radiotherapy); LAR (low anterior resection); APR (abdominoperineal resection);

Perioperative mortality was 3.4% with two patients dying after anastomotic leakage and subsequent peritonitis, one patient dying from sepsis and one patient dying from postoperative hemorrhage. Postoperative complications were observed in 22.8% of the patients with anastomotic leakage (n = 10) and wound infection (n = 9) the most frequent complications (Table 3). Re-operation due to perioperative complications was required in 18 patients.

**Table 3:** Perioperative complications

Perioperative complication	Number of patients (%)
Death	4 (3.4)

Anastomotic leakage	10 (8.5)
Wound infection	9 (7.6)
Wound dehiscence	3 (2.5)
Ileus	2 (1.7)
Postoperative hemorrhage	2 (1.7)
Colo-cutaneous fistula	1 (0.8)

Administration of adjuvant chemotherapy was indicated in 81 patients with pathological stage UICC II - IV. However, chemotherapy was delivered in only 61/81 (75.3%) of these patients. 35 patients were treated with 5-FU and folinic acid, 15 patients with FOLFOX4 and 11 patients with other protocols. Pre-treatment performance status was significantly better in the subgroup treated with adjuvant chemotherapy compared to the subgroup not receiving adjuvant chemotherapy (p = 0.002). Additionally, postoperative complications were observed more frequently in the subgroup, which was not treated with adjuvant chemotherapy (41% versus 18%).

Twelve patients suffered from late toxicity > grade II, with late anastomotic leakage and abscess formation requiring surgery (Grade IV, n = 4, 3.4%) and small bowel ileus (grade III, n = 1, 0.8%; grade IV, n = 3, 2.5%) the most frequent toxicities (Table 4). This resulted in an actuarial 5-year rate of late toxicity > grade II of 12% (95% confidence interval 5% to 19%). Median interval between primary surgery and surgery for anastomotic leakage was 34 months ranging between 7 months and 64 months. No death because of late toxicity was observed.

**Table 4:** Severe late toxicity grade > II CTCAE v3.0

Late toxicity	Number of patients (%)	
	Grade III	Grade IV
Late anastomotic leakage		4 (3.4)
Small bowel ileus	1 (0.8)	3 (2.5)
Chronic diarrhea	3 (2.5)	
Anal incontinence		1 (0.8)

### Survival and recurrence

**Figure 1:**
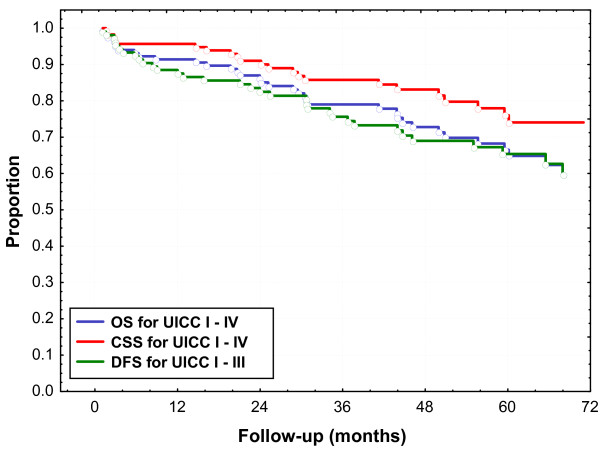
**Overall survival (OS), cancer specific survival (CSS) and disease free survival (DFS)**.

Median follow-up was 46 months for all patients and 53 months (range 3.2 to 93) for living patients; follow-up for living patients was shorter than 3 years for 19 patients. The five-year OS rate was 67% for all patients and 70% after exclusion of UICC stage IV (Fig. 1). OS was not statistically significant for UICC stages I to III: five-year OS was 81%, 74% and 59% for UICC stage I, II and III, respectively. Five-year OS was 70% and 50% for UICC stage II to IV when adjuvant chemotherapy was given or not (p = 0.12), respectively.

Five-year CSS was 76% for all patients (Fig. 1). Thirty-six patients died during follow-up and cause of death was progression of rectal cancer (n = 19), intercurrent disease (n = 14), and unknown (n = 3). Time to death was median 15 months and 31 months for patients dying from rectal cancer or from intercurrent disease, respectively; all patients, who died from intercurrent disease, had no evidence of recurrent tumor. Five-year DFS for all patients excluding UICC stage IV was 65% (Fig. 1). Five-year DFS was 78%, 71% and 52% for UICC stage I, II and III, respectively.

**Figure 2:**
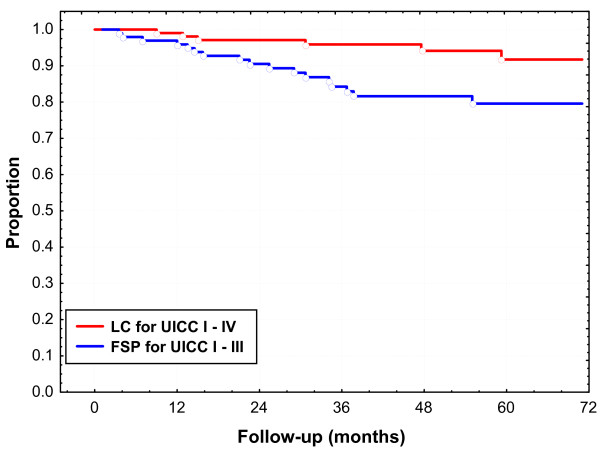
**Local control (LC) and freedom from systemic progression (FSP)**.

A total of six local recurrences were observed resulting in a five-year LC of 92% (Fig. 2). Local recurrence developed prior to (n = 3), simultaneously (n = 1) and after (n = 2) systemic progression of disease. Pathological T stage was pT2 (n = 1), pT3 (n = 4) and pT4 (n = 1). Two of six local recurrences developed after R1 resection. Local recurrences were observed between 9 and 59 months. Five recurrences developed in the presacral region, which had been routinely included into the PTV; one lymph node metastasis in the groin was considered as local recurrence.

Five-year FSP was 75% for all patients and 80% for patients after exclusion of stage UICC IV (Fig. 2); median time to systemic progression was 23 months.

## Discussion

Preoperative short-course radiotherapy with five fractions of 5 Gy is well established to increase survival after conventional surgery [4] and to increase local control after TME surgery [5,8]]. This study modified the short course regime in two aspects: radiotherapy was delivered with twice daily fractions of 2.9 Gy to a total dose of 29 Gy in one week immediately prior to surgery and adjuvant chemotherapy was planned for UICC stage ≥ II.

After a sufficiently long follow-up of median 46 months, five-year OS was 67% in our patient cohort. This compares well to data from the literature. The Swedish Rectal Cancer Trial reported a five-year OS of 58% for the group treated with preoperative radiotherapy despite a more favourable distribution with regard to tumor stages [4]. The distribution of the UICC stage in the Dutch TME trial was similar to our study and five-year OS was 64% after preoperative radiotherapy and quality controlled TME surgery [8]; that study had a higher proportion of patients with low tumor location. The recently published MRC CR07 trial reported a five-year OS of 70% [9]; adjuvant chemotherapy with 5-FU and leucovorin was allowed and given to 40% of the patients after preoperative radiotherapy and TME surgery. After preoperative long-course radiochemotherapy in the German Rectal Cancer Trial, five-year OS was 76% [3].

Similar to the two randomized trials using short-course preoperative radiotherapy followed by TME surgery [4,5], local control was high in the present study with a five-year local control rate of 92%. Six local recurrences were observed and R1 resection had been performed in two of these six patients. Interestingly, two local recurrences developed late: 4 and 5 years after treatment. This observation of late local recurrences after more than 3 years is in agreement with Peeters et al. [8], whereas late local recurrences were rare in the Swedish Rectal Cancer Trial [10] and the MRC CR07 trial [9]. Despite high rates of local control, about 25% of the patients suffered from systemic progression of disease resulting in DFS of 65% after five years. This contrast of low rates of systemic control on the one hand and excellent local control on the other hand, implicates the need for more effective systemic chemotherapy.

Adjuvant 5-FU based chemotherapy was planned for UICC stage ≥ II in our study; however, only 74% of these patients actually received chemotherapy. Lower performance status and more frequent postoperative complications prevented administration of chemotherapy in a quarter of the patients. Despite the planned use of bolus 5-FU and LV chemotherapy, 40% of the patients were treated with different regimes, most frequently FOLFOX, after the MOSAIC trial had been published [11]. The difficulty of postsurgical (radio-) chemotherapy has been described by Sauer et al.: 89% and 50% of the patients in the preoperative and postoperative radiochemotherapy group received full dose chemotherapy, respectively [3]. Whether the difference in the proportion of patients with adjuvant chemotherapy between our study and long-course preoperative radiochemotherapy in the CAO/ARO/AIO-94 trial is related to the short-course radiotherapy remains speculative. However, acute toxicity during short-course radiotherapy was very mild and perioperative morbidity was similar to the preoperative arm in the German study, which does not support the hypothesis of a detrimental effect of short-course radiotherapy. Differences in patient characteristics and differences in the aggressiveness to perform adjuvant chemotherapy are most likely to explain this difference.

Our own data do not allow the conclusion that adjuvant chemotherapy improves clinical outcome but clearly prove its feasibility with favourable results. Five-year OS was 70% and 50% when adjuvant chemotherapy was given or withheld, respectively, but this difference did not reach statistical significance. The patient number in our study is certainly too small for such subgroup analysis. Assuming a difference between the two groups, this difference could result from improved outcome after adjuvant chemotherapy or from differences in patients' performance status and postoperative morbidity.

Although patients with rectal cancer clinical UICC stage I were excluded from this study, pathological UICC stage I was observed in 27% of the patients. Staging of the T and N status was based on endosonography, which was considered as best practice at the time this study protocol was developed [12-14]. Endorectal ultrasound was performed in 95% of our patients. However, the inaccuracy of endosonography in daily clinical routine is well known and the overstaging rate of about 25% compares well to data from other studies. The German Rectal Cancer Trial comparing preoperative with postoperative radiochemotherapy found a 18% rate of UICC stage I patients in the group treated with postoperative radiochemotherapy [3]. Widder et al reported 33% of the patients with UICC stage I disease after short-course preoperative radiotherapy [15]. This suboptimal performance of endosonography may be caused by inconsistent operator experience [16] or prior biopsy of the cancer, which was shown to result in decreased accuracy of endosonography staging [17]. Recently, pelvic magnetic resonance imaging (MRI) showed excellent accuracy for evaluation of the extramural depth of tumor invasion [18]; however, staging of the N status and differentiation between clinical stage UICC I and II is difficult even with MRI imaging [19,20]. These uncertainties of preoperative staging, where overstaging of the T stage was the most frequent finding in pathological analysis, are considered strong arguments for preoperative short course radiotherapy, where downstaging is usually not observed and pathological staging can then be used for selection of patients, which might benefit from adjuvant chemotherapy.

Perioperative mortality was 3.4% and anastomotic leakage was the most frequent complication with an incidence rate of 8.5% in our study. This compares well with published data in literature, which shows that preoperative short course radiotherapy with multi-field radiation techniques is not associated with an increased risk of acute perioperative complications [21,22]. However, patients need to be informed about the increased risk of late toxicity and complications. Increased bowel frequency, incontinence, urgency, and emptying difficulties have been described by Dahlberg et al. after radiotherapy in the Swedish Rectal Cancer Trial [23]. Peeters et al. reported similar findings with increased rates of fecal incontinence, anal blood loss and lower satisfaction with bowel function in irradiated patients compared with patients who underwent TME alone [24]. The most frequent severe late toxicity was late anastomotic leakage with abscess formation, which was observed in 4 patients (3.4%). This rather high rate of late anastomotic leakage has not been described in the literature. In contrast, the overall rate of late toxicity > grade II toxicity was 10%, which is comparable to other studies on preoperative short-course radiotherapy and long-course radiochemotherapy: small bowel ileus was observed in 3.4%, chronic diarrhoea in 2.5% and anal incontinence in only 1% of the patients. Similar to experiences from other malignancies in the pelvic region [25-28], the use of more conformal radiotherapy treatment planning techniques could reduce toxicity in future protocols.

Our fractionation protocol was designed for isotumor efficacy with simultaneously reduced risk of late toxicity compared to five fractions of 5 Gy: radiotherapy was delivered in twice daily fractions of 2.9 Gy to a total dose of 29 Gy with a least a six hours interval between the daily fractions. Biological effective doses (BED) were calculated for the irradiation protocols 5 × 5 Gy, 25 × 1.8 Gy, 28 × 1.8 Gy and 10 × 2.9 Gy and results for the tumor (α/β = 10 Gy) and organs-at-risk (α/β = 3 Gy) are shown in table 5. The anti-tumor efficiency was largest for conventionally fractionated 50.4 Gy with no difference between the other three protocols if overall treatment time is considered in BED calculations. In contrast, large differences in effective doses to organs-at-risk were observed with lowest risk of late toxicity for our protocol of 10 × 2.9 Gy. These theoretical calculations suggest the best therapeutic ratio for the short-course protocol with twice daily irradiation. Widder et al. reported a similar concept of short-course preoperative radiotherapy with twice daily fractions of 2.5 Gy to a total dose of 25 Gy within one week [15]. The authors reported a high local control rate (98% after 4 years) along with low rates of toxicity. However, it should be kept in mind that theoretical anti-tumor efficiency is significantly reduced in that fractionation protocol. A Polish group reported a moderately low α/β value of 5 Gy for rectal cancer [29], which would further support hypo-fractionated protocols. However, this low α/β value is based on retrospective single-institution data and the confidence interval was large ranging between -0.1 Gy to 10.3 Gy.

**Table 5:** Calculation of biological effective doses (BED) for the rectal tumor (α/β = 10 Gy) and late normal tissue toxicity (α/β = 3 Gy)

	29 Gy (10 × 2.9 Gy)	25 Gy (5 × 5 Gy)	45 Gy (25 × 1.8 Gy)	50.4 Gy (28 × 1.8 Gy)
**Tumor OTT (α/β = 10 Gy)**	37.4	37.5	37.5	42.1
**Tumor (α/β = 10 Gy)**	37.4	37.5	53.1	59.5
**Normal tissue (α/β = 3 Gy)**	57.0	66.7	72.0	80.6

Overall treatment time (OTT) was considered (first row) or not (second row) at calculation of BED doses to the tumor.

Preoperative short-course radiotherapy is certainly not the optimal approach for all patients with rectal cancer, and this was considered in the inclusion criteria of this study. Neoadjuvant radiochemotherapy outside this trial was chosen instead of preoperative short-course radiotherapy if the surgeon and radiation oncologist expected that downstaging could 1) improve complete resectability in patients with cT4 tumors or 2) achieve sphincter preservation in patients with low tumor location. Patients with invasion of the sphincter or very low tumor location, where no sufficient downstaging for sphincter preservation after neoadjuvant radiochemotherapy was assumed, were eligible. Despite this trial was limited to potentially resectable patients and only one patient with cT4 disease was included, a 5.1% incomplete resection rate was observed. It is speculative, but MRI could be used for selection of patients with a small predicted circumferential margin [30]: these patients could benefit from long-course radiochemotherapy instead of short-course radiotherapy.

## Conclusions

Preoperative short course radiotherapy with twice daily fractions of 2.9 Gy to a total dose of 29 Gy combined with adjuvant 5-FU based chemotherapy for rectal cancer UICC stage ≥ II was well tolerated with low rates of acute and late toxicity. Treatment resulted in favourable local control and survival. High rates of systemic progression require intensification of systemic chemotherapy.

## Competing interests

The authors declare that they have no competing interests.

## Authors' contributions

All authors read and approved the final manuscript.

MG: acquisition of data and data analysis, statistical analysis, writing and drafting of the manuscript.

JW: conception and design of the study, acquisition of data and data analysis.

AT: acquisition of data and data analysis.

DW: acquisition of data and data analysis.

AT: conception and design of the study.

GM: acquisition of data.

MS: conception and design of the study.

MF: conception and design of the study.
